# Are providers prepared for genomic medicine: interpretation of Direct-to-Consumer genetic testing (DTC-GT) results and genetic self-efficacy by medical professionals

**DOI:** 10.1186/s12913-019-4679-8

**Published:** 2019-11-25

**Authors:** Scott P. McGrath, Nephi Walton, Marc S. Williams, Katherine K. Kim, Kiran Bastola

**Affiliations:** 1 School of Interdisciplinary Informatics, University of Nebraska at Omaha, 1110 S 67TH St., Omaha, 68182 NE USA; 2 Genomic Medicine Institute, Geisinger, 100 N. Academy Ave., Danville, 17822 PA USA; 3 Betty Irene Moore School of Nursing, UC Davis, 2570 48th St., Sacramento, 95817 CA USA

**Keywords:** Precision medicine, Commercial genetics, Direct-to-consumer genetic testing, Primary care, Genetic counseling

## Abstract

**Background:**

Precision medicine is set to deliver a rich new data set of genomic information. However, the number of certified specialists in the United States is small, with only 4244 genetic counselors and 1302 clinical geneticists. We conducted a national survey of 264 medical professionals to evaluate how they interpret genetic test results, determine their confidence and self-efficacy of interpreting genetic test results with patients, and capture their opinions and experiences with direct-to-consumer genetic tests (DTC-GT).

**Methods:**

Participants were grouped into two categories, genetic specialists (genetic counselors and clinical geneticists) and medical providers (primary care, internists, physicians assistants, advanced nurse practitioners, etc.). The survey (full instrument can be found in the Additional file 1) presented three genetic test report scenarios for interpretation: a genetic risk for diabetes, genomic sequencing for symptoms report implicating a potential HMN7B: distal hereditary motor neuropathy VIIB diagnosis, and a statin-induced myopathy risk. Participants were also asked about their opinions on DTC-GT results and rank their own perceived level of preparedness to review genetic test results with patients.

**Results:**

The rates of correctly interpreting results were relatively high (74.4% for the providers compared to the specialist’s 83.4%) and age, prior genetic test consultation experience, and level of trust assigned to the reports were associated with higher correct interpretation rates. The self-selected efficacy and the level of preparedness to consult on a patient’s genetic results were higher for the specialists than the provider group.

**Conclusion:**

Specialists remain the best group to assist patients with DTC-GT, however, primary care providers may still provide accurate interpretation of test results when specialists are unavailable.

## Background

Availability of Direct-to-Consumer genetic testing (DTC-GT) has grown in the United States over the past decade, and they have recently started to cross over from a niche product to something with mainstream appeal [1]. Interest in DTC-GTs, which are available online from company websites and major retailers like Amazon, have been accelerating. In fact, the DTC-GT kit sold by 23andMe was one of the top 5 best sellers for Amazon’s Black Friday sale in 2017 [2]. The number of DTC-GT kits sold offering ancestry results has doubled since 2017, with over 12 million kits sold [3]. Despite the growing interest and use of DTC-GT kits, only 10-20% of DTC-GT customers had shared their genetic test results with primary care physicians [4, 5]. Previous studies have shown that the typical consumer of DTC-GTs held both higher levels of education and pay in contrast to the average American citizen [4, 6]. As the consumers of these tests continue to shift from hobbyists to the general public, it is likely that more individuals will request assistance in interpreting their results. Previous research has shown that physicians feel ill prepared to educate patients about genetic test results [7–11]. Presently, there is a knowledge gap in how accurately primary care providers can interpret and communicate genetic test results to patients in comparison to genetic specialist. There have been some previous studies which have focused on primary care providers and genetics [12–15] but our study is novel in that it samples a larger cohort of genetic specialists (*n*=165) and non-genetic providers (*n*=99). This allows us to provide more robust measurements of interpretation differences between genetic specialists and non-genetic specialists compared to prior studies. Globally, patients from Australia, Germany, United States, and elsewhere have expressed a preference for some form of regulation of DTC-GT and for physician assistance in interpreting this kind of data [16–18]. Purchasing DTC-GT is an elective process for someone to opt-in to, inputting genomic data into patient records will switch gears to an opt-out process. We are approaching a period of time where physicians encountering patients bringing in their DTC-GT reports will shift to genomic results appearing in the majority of all their patient’s EHRs, thanks in part to projects like the Precision Medicine Initiative. Studies like the Clinical Sequencing Exploratory Research Consortium (CSER) [19] and the electronic MEdical Records and GEnomics (eMERGE) network have been working on integrating genomic medicine into clinical care for several years now. eMERGE has been combining EHRs and genomics to improve discovery and patient care since its launch in 2007 [20, 21]. Now in its third round of funding, eMERGE is generating clinically actionable results to return to patients in the EHR [22, 23] The CSER Genetic Counseling Working Group (GC WG) was launched in 2012 and is dedicated to assisting genetic counselors with challenges encountered with genomic medicine (genomic education, results disclosure, and other issues) [19]. As work progresses on these issues, the line separating clinical genetic testing and DTC-GT is becoming harder to define [24]. Recognizing the current and future need for integration of genomic data into the EHR, the two largest EHR vendors, Epic and Cerner have recently enabled their systems to store structured genomic data in the patient record [25, 26].

The United States based Precision Medicine Initiative (PMI) established $215 million to help actualize precision medicine in 2015 [27]. Patient genetic data for individuals, information about their lifestyle, and environmental data would be directly incorporated into diagnosis and treatment recommendations in addition to the standard family and patient histories currently found in medical records. Precision medicine requires the collection of robust, multifaceted biomedical data sets; therefore, a major task of the initiative is to recruit a million volunteers to help capture this necessary data. A wide range of data types would be collected from this cohort, including genetic and microbiome sequencing, lifestyle data, metabolites, and information from wearable sensors. As of June 2017, beta testing for this program, now known as All of Us, has begun [28]. One component of the PMI initiative is to incorporate more patient genetic data into EHRs. This action should facilitate data analysis and inter-operability, which means medical providers need to be able to accurately and confidently interpret genetic information for patients.

As we see a shift from DTC-GT over to more genetic results piped into the EHR, clinicians will be increasingly called upon to interpret and act on results. With the growing consumer interest in DTC-GT [24] there is a shortage of trained specialists needed to meet this demand [29]. Thus, primary care physicians will likely be asked to interpret DTC-GT results with increasing frequency. Some primary care providers have already begun helping their patients interpret genetic test results [30]. Success will be defined by the ability of these physicians to properly communicate results and risks identified from genetic and genomic tests back to their patients. Many DTC-GT results relate to small risk changes for common diseases and pharmacogenetics, which may be more appropriately interpreted as part of primary care and may not require a visit to a genetics specialist. Failure in this task will erode the fundamental clinical value proposed by precision medicine. The complexity of these tests is one of the reasons the American College of Medical Genetics and Genomics has recommended that genetics experts be made available for patient test result consultations [31]. Based off professional memberships and certification in the United States, there are only 4,244 genetic counselors [32] and 1,302 clinical geneticists [33] currently employed across the nation, numbers that may be insufficient to meet the potential future demand. Genetic counselors have advanced training in medical genetics and counseling to help patients with education and interpreting genetic test results [34] and are often the first point of contact for a patient with a suspected genetic condition or test result. Clinical genetics is a certification offered to physicians by the American Board of Medical Genetics and Genomics. Clinical geneticists are trained to diagnose and treat genetic conditions and work cooperatively with genetic counselors across the entire range of genetic disease [35]. Increased market demand may lead to more genetic counselors and medical geneticists entering the field; however presently there is a shortage of genetic counselors engaged in direct patient care, which may not be fully resolved until 2024-2030 based off current projections [29].

As precision medicine and genetic testing expand, primary care providers may be increasingly relied upon to interpret results for patients. There are efforts underway to help adapt physician training in anticipation of the genomic element of precision medicine [36], but the formal systems and tools to assist with this task are still being developed [37, 38]. Establishing a benchmark on the ability of physicians to interpret these results will aid in the development of these tools. However, there has been no study examining if medical providers can accurately interpret DTC-GT results for patients when compared to specialists, or whether they feel prepared to provide DTC-GT interpretation to patients. Therefore, the purpose of this study was to understand healthcare providers’ genetic self-efficacy, their ability to interpret genetic results, and establish how prepared they feel to accomplish this task. We adapted with permission a survey instrument [6] that was designed to understand consumer comprehension of DTC-GT. Therefore we polled primary care providers and genetic specialists on the following aims: how each group interprets genetic test results; determine their confidence and self-efficacy of interpreting genetic test results with patients; and capture their opinions and experiences with DTC-GT. Our hypothesis is that there is a discernible difference between the two groups in interpretation of DTC-GT results and how prepared they feel to undertake this task.

## Methods

The online survey was comprised of 33 questions. This survey was modeled with permission from the Ostergren study, which evaluated consumer comprehension of DTC-GT [6]. We selected two of the four scenarios presented in Ostergren’s survey and added a third case that represented a more complicated clinical test with a patient facing report to assess their performance in a more complicated scenario. Face validity for our study was achieved by having two subject matter experts review the questions for accuracy and have a separate expert on question construction review the survey instrument. There were three phases to the survey. The first phase collected demographic information and assessed genetic self-efficacy. The second phase tested the participant’s ability to accurately answer questions on three genetic interpretation scenarios. The final phase collected their opinions on the trustworthiness of DTC-GTs and how prepared they felt to discuss these results with patients. A free text box for feedback was offered at the end of the survey. This latter data was not qualitatively analyzed, but it did provide some contextual information for the results.

### Targeted populations

**Fig. 1:**
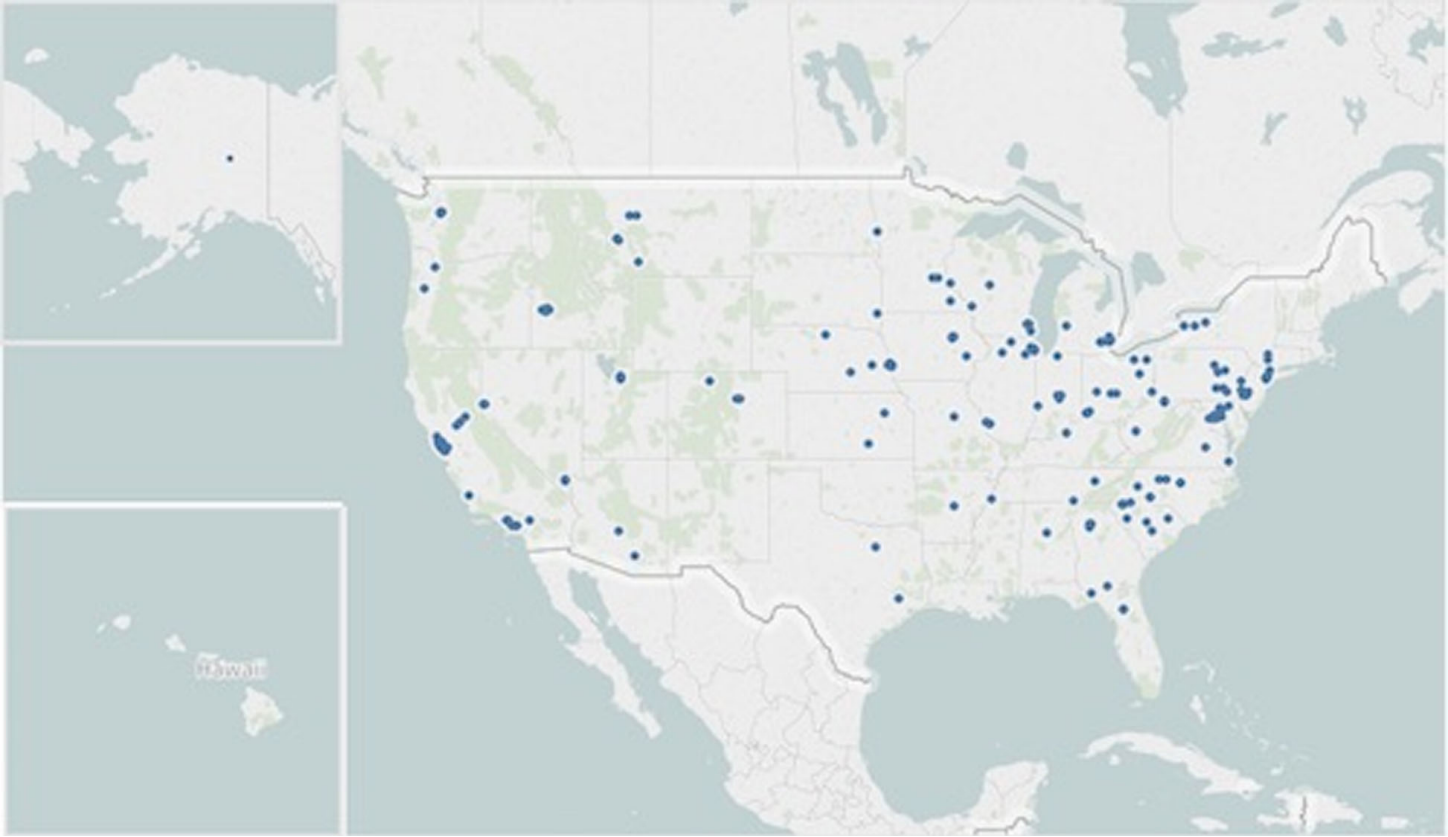
Map of participants across the United States, built with Tableau version 2019.1. Additional details and views can be found at https://public.tableau.com/views/Mapofparticipants/Dashboard1

Participants were recruited from two populations: “Specialists” (genetic counselors and clinical geneticists), and “providers” (primary care healthcare providers). We define primary care providers (PCP) following the definitions outlined by the Agency for Healthcare Research and Quality (AHRQ) and US Health Resources and Services Administration, which state that “medical specialties that could meet the criteria for a PCP include general and family medicine, general pediatrics, general internal medicine, and geriatrics.” [39]. The AHRQ also includes nurse practitioners and physician assistants in their definition of primary care providers [40]. Specialists were recruited through the National Society of Genetic Counselors and direct email solicitation. Primary care healthcare providers were recruited through email, professional societies (SGIM, AMIA, AAPA, AANP), web fora (Reddit), social media (Twitter, Facebook, and Fig. 1). Primary care providers included a variety of specialties including internists, family medicine, nurse practitioners, physician assistants and others. The full breakdown of providers polled is defined in Table 1. Group selection was made on the basis that one group is highly specialized in interpreting results and educating patients on this topic, and the other for their high probability of being the first medical provider patients ask to assist in interpreting DTC-GT results. These two groups are hereto referred to as the specialist group for those with certification in genetics-based practice (genetic counselors and clinical geneticists) and providers for the primary care providers.

**Table 1:** Participant Demographics (*n* = 264)

	Total	Provider	Specialist
Mean age ± SD (range), years	39.9 ±13.7 (21-81)	48.9 ±14 (21-81)	34.6 ±10.3 (23-75)
Years practicing	12.4 ±11.8 (1-50)	18.7 ±12.7 (1-50)	8.6 ±9.2 (1-47)
Gender			
*Male*	19.7%	39.4%	7.8%
*Female*	80.3%	60.6%	92.2%
Race			
*White*	90.1%	88.9%	90.9%
*Asian*	5.0%	5.1%	4.9%
*Black or African American*	0.7%	2.0%	0.0%
*Two or more races*	1.2%	1.0%	1.2%
*Did not wish to self identify*	0.7%	1.0%	0.6%
*Ethnicity*			
*Hispanic or Latino*	2.3%	2.0%	2.4%
Medical Specialty			
*Internal Medicine*	9.5%	25.3%	
*Primary care (MD)*	6.8%	18.2%	
*Nurse Practitioner*	6.1%	16.2%	
*Family medicine (MD)*	5.7%	15.2%	
*Physician Assistant*	5.3%	14.1%	
*Other∗*	2.3%	6.1%	
*Emergency Medicine*	1.9%	5.2%	
*Genetic Counselor*	61.0%		97.6%
*Clinical Geneticists*	1.5%		2.4%
Work Environment			
*Hospital*	42.8%	26.3%	52.7%
*Clinic*	14.0%	15.2%	13.3%
*Group Practice*	9.8%	20.2%	3.6%
*Integrated Healthcare Delivery System*	8.7%	13.1%	6.1%
*Laboratory*	6.4%		10.3%
*Employed Physician Practice*	6.4%	12.1%	3.0%
*University Medical Center*	3.0%	4.0%	2.4%
*Academia*	1.9%	3.0%	1.2%
*Solo Practice*	1.5%	1.0%	1.8%
*Other*	1.5%	2.0%	1.2%
*Clinical Research*	1.1%		1.8%
*Health Center*	1.1%	3.0%	
*Industry*	0.8%		1.2%
*Telecommute*	0.8%		1.2%

^*^Medical oncology, clinical research, endocrinology, pediatric sports medicine, and infectious diseases

### Genetic self-efficacy

Genetic self-efficacy was measured on a six-point Likert scale (1 = Strongly agree to 6 = Strongly disagree) across five questions. Following the design of the Ostergren study, the self-efficacy questions were adapted from the six-item measure of genetic self-efficacy by Kaphingst [41, 42]. The only modification to Kaphingst’s instrument was the exclusion of the first question: “I am able to understand information about how my genes can affect my health”. Strong internal consistency across the five questions was shown with a Cronbach’s alpha of 0.97 for the specialists and 0.92 for the providers.

### Scenario testing

Three test scenarios were developed to model three aspects of DTC-GT results: disease risk, genetic symptoms testing, and drug response. Each scenario question included a best interpretation answer, and an interpretation score was generated by calculating the total number of correct over the three scenarios for a scale of 0-6. Scores were interpreted in aggregate, with no weighting, as has been done in prior studies [4–6, 43]. Scenarios 1 and 3 are identical to the ones used in the Ostergren study. The first scenario was modeled off a disease risk report from the company 23andMe. It presents a DTC-GT result for a 35-year old male, classified as obese, and his genetic susceptibility for diabetes (3 questions). The third scenario was a genetic drug response result from the company Pathway Genomics. It presents risk level for statin-induced myopathy for a male (age unspecified) taking the statin drug simvastatin to control his cholesterol (1 question). The second scenario was a novel scenario constructed using the patient generated report from a genomic test tool, COMPASS™, developed at Geisinger [44] which presents the result of a clinical genetic test. COMPASS™generates two different reports: a patient focused report and a provider focused report. The second scenario was constructed using results from the patient version of the report. The scenario presented the symptoms report for a 48-year old woman with three children. In order to help diagnose her symptoms, a genetic sequencing test was ordered. From that test, *DCTN1* is highlighted as noteworthy, because a variant of this gene can cause HMN7B: distal hereditary motor neuronopathy type VIIB, which matches some of her symptoms (2 questions).

### Opinion analysis

Opinions of the two groups were compared across three different categories: patient concern, DTC tests, and genetic self-efficacy. For the patient concern questions, survey participants were asked to rate the level of concern the patient should have based off the diabetes and HMN7B test results. In the DTC category, they were asked to rank how trustworthy were each scenario’s information (5 point Likert, 1 = Highly Trustworthy, 3 = Neutral, 5 = Highly Untrustworthy), which we refer to as the *trustworthiness* variable. The second half of the DTC test category asked how prepared they personally felt to discuss DTC-GT results with patients (5-point Likert, 1 = Well prepared, 3 = Neutral, 5 = Very unprepared), which we refer to as the *preparedness* variable. Genetic self-efficacy scores, or their innate abilities to understand and discuss genetics, were aggregated from 5 questions with a 1 equaling a “strongly disagree” and a 6 equaling a “strongly agree”.

### Data analysis

SPSS version 22 was used for statistical analysis and Tableau Desktop version 2018.1.4 was used to construct data visualizations. Cronbach’s alpha was used to examine the internal consistency of participant’s answers on the self-efficacy questions. A low Cronbach’s alpha score would indicate contradicting answers to the questions; for example, stating high confidence in their ability to explain to others about how genes affect one’s health, but a low confidence in knowledge on how genetics may influence risk for disease. Chi-square analyses were used to compare interpretation scores from the three scenarios. We looked at two different analyses, one to analyze total scores from all six-interpretation scores, and another to inspect each question individually.

Binomial logistic regression was performed on seven variables to assess the effects of grouping (specialist or provider), age, number of years practicing medicine, prior DTC test result consultations (Y/N), trustworthy variable, prepared variable, and average genetic self-efficacy scores on the likelihood of several outcomes (total interpretation scores, and scores on individual questions). Linearity of the continuous variables with respect to the logit of the dependent variable was assessed via the Box-Tidwell procedure [45]. A Bonferroni correction was applied using all seven terms in the model resulting in statistical significance being accepted when p <.007 [46]. Based on this assessment, six continuous independent variables were found to be linearly related to the logit of the dependent variable. One variable required transformation: years of practice. A “reflect and inverse” transformation was performed due to extreme left skew to the data, driven by a high percentage of participants who listed only 1 year in practice. Multicollinearity among independent variables was assessed using the standard errors for the *β* coefficients with a threshold of >2.0 to detect potential outliers. Cases where the studentized residual with a values of exceeding 2.75 standard deviations and were removed are documented. The interpretation sum score was dichotomized into higher and lower comprehension through a median split procedure, with lower comprehension (below or equal to the median of 5; *n* = 208) or higher comprehension (6 out of 6; *n* = 56) groups. The regression method used was a stepwise backward logistic regression (Backward: LR) for removing variables in the model. Backward LR starts with all variables and uses the likelihood ratio test to determine which variables are removed to improve the model.

## Results

### Demographics

427 surveys were initiated and 264 completed (62% completion rate) with a response rate of 9.1% (427 out of 4,697). Gender ratios were significantly skewed (*p* <.01) towards females both in the total (19.7% male and 80.3% female) and between groups. This may be due to sampling professions which are majority female: genetic counselors (96% of the field is female [47]), nurse practitioners (88% female [48]), and physician assistants (63% female [49]). Combined, these three groups accounted for 72.4% of the surveyed professions. Over 90% of respondents identified as white, non-Hispanic or Latino.

### Result interpretation

**Fig. 2:**
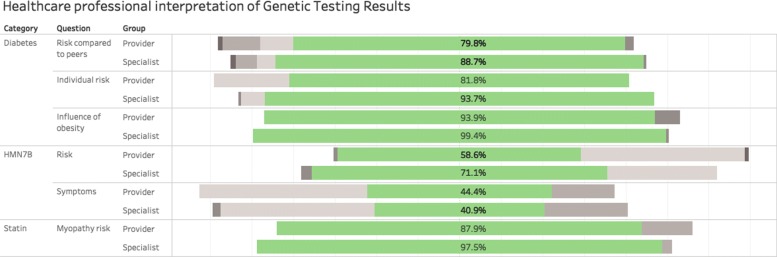
Interpretation rates by group across the six scenarios. The proportion that had a correct interpretation is depicted in green, while selection of the other offered answers are in shades of grey. Additional details and views of the graph can be found at https://public.tableau.com/views/TestingInterpretation/Dashboard1

A comparison of scenario interpretation scores between the two groups found the providers averaged 74.4% correct compared to the average of 83.4% by the specialists (*p* <.001). Both groups performed better in scenarios 1 and 3, while lower in scenario 2. Figure 2 provides additional details on the how each group interpreted the provider and specialists scored each individual case.

### Opinion analysis

**Fig. 3:**
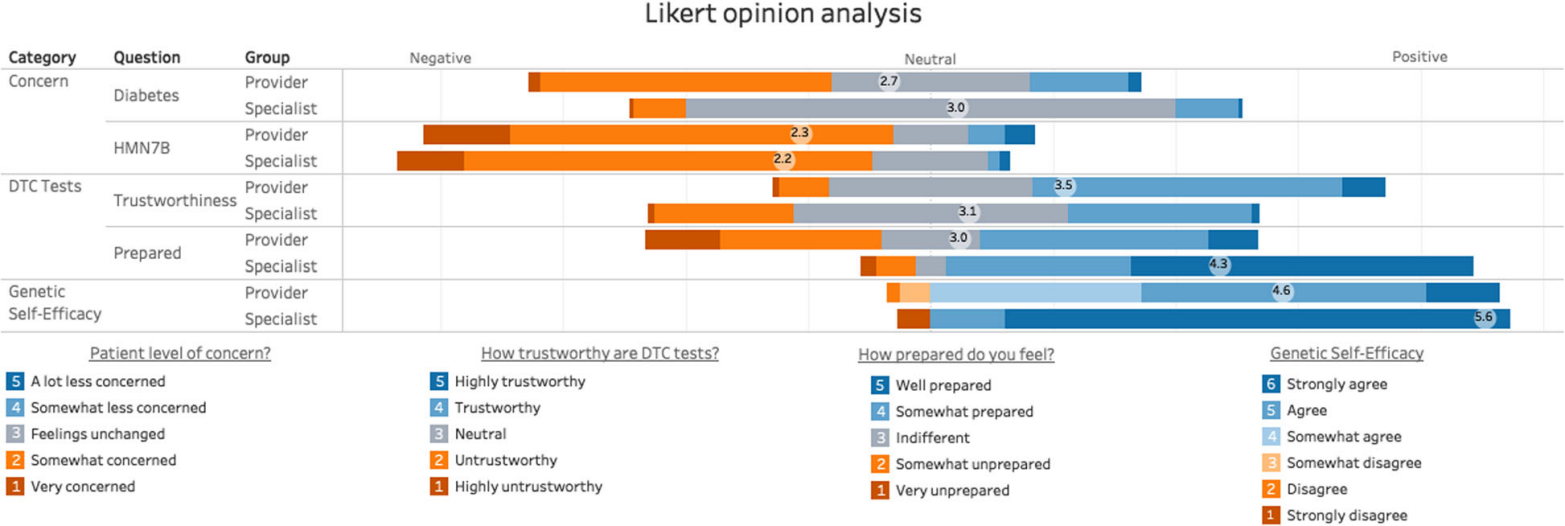
Participants opinion analysis. Additional details and views can be found at https://public.tableau.com/ views/Surveylikert/Dashboard3

The results presented in Fig. 3 shows the genetic specialists had high self-efficacy in their ability to discuss genetic results with patients (*X* ¯ = 5.6 ±1.15) and a Cronbach’s alpha test showed strong internal consistency (0.974). Provider’s self-efficacy was slightly lower (*X* ¯ = 4.6 ±0.84, Cronbach’s alpha = 0.918). Genetic specialists were more confident in their ability to discuss DTC results with patients (*X* ¯ = 4.3 ±1.0) compared to the providers (*X* ¯ = 3.0 ±1.2), which was statistically significant (*p* <.001). When asked if they had encountered patients who had brought in their personal DTC-GT results, 57.9% of the specialists said yes (with an average number of 5 patients) compared to the 17.2% of providers (averaging 2.5 patients).

### Regression analysis

Several binomial regression models were run from the results. The first case was evaluating everyone who scored 6 out of 6 in the interpretation tasks (Table 2). The logistic regression model was statistically significant, *χ*^2^(7) = 23.31, *p* <.001. The model explained 16.9% (Nagelkerke *R*^2^) of the variance in test interpretation and correctly classified 81% of cases. Of the eight-predictor variables in the model, only four were statistically significant: age, those who were either neutral about or trusted the DTC reports, and prior DTC test consultations (as shown in Table 1). The area under the ROC curve was.729 (95% CI,.659 to.80), which is an acceptable level of discrimination according to Hosmer [50]. Those with prior exposure to DTC test from patients were 2 times more likely to properly interpret the scenarios than those who have not had patients bring in their results. Those who ranked their level of trust as neutral or trustworthy were 3.2 to 3.8 times respectively more likely to properly interpret the cases than those with stronger opinions or those who distrusted the reports. Increasing age was associated with a small decreased likelihood of properly interpreting the scenario by a factor of 1.04.

**Table 2:** Summary of logistic regression for scoring highly (6/6) on interpretation tasks (n = 253*)

Variables	B	S.E.	Wald	Df	Sig	Odds Ratio	95% C.I. for Odds Ratio	
							Lower	Upper
Age	-0.04	0.02	6.12	1	0.01	0.96	0.93	0.99
*Trustworthy*			5.98	4	0.2			
Highly Untrustworthy	-19.25	22353	0	1	1	0	0	.
Highly Trustworthy	-18.32	13481	0	1	1	0	0	.
Neutral	1.18	0.56	4.55	1	**0.03**	3.27	1.1	9.69
Trustworthy	1.35	0.56	5.84	1	**0.02**	3.87	1.29	11.62
DTCCustomers(1)	0.71	0.35	3.99	1	**0.046**	2.03	1.01	4.06
EfficacyAve	-0.24	0.18	1.7	1	0.19	0.79	0.55	1.13
Constant	-0.89	0.8	1.22	1	0.27	0.41		

**Note**: 6 participants from the original 264 did not answer the trustworthy question and thus were not included in this regression; 5 participants were removed as outliers for having studentized residuals >2.75

We also ran a subsequent analysis on the individual scenario questions. When looking for predictors of who correctly interpreted the HMN7B question 1 (Erin’s chances of HMN7B), the model was statistically significant, *χ*^2^(9) = 42.62, *p* <.001. The model explained 21% (Nagelkerke *R*^2^) of the variance test interpretation and correctly classified 71% of cases. Age (odds ratio 1.04, p =.03) and Trustworthy (odds ratio 67.99, *p* =.01) were again present, but prior exposure to DTC-GT (factor of 3.7, p =.046) was replaced by if they had indicated a neutral opinion assessing how prepared they felt to assist patients with their genomic results. The HMN7B scenario presented a unique case that is explored in the discussion.

## Discussion

One of the goals of precision medicine is to include genomic data in patient records. With the limited number of specialists in the field, primary care providers will be the most likely candidates to meet demand as precision medicine data becomes more widely available. However, there is a knowledge gap on how well healthcare providers interpret genomic test data in comparison to those with focused training in genetics. Prior studies have shown that primary care physicians can manage genetic test results appropriately [12, 13] and they have some level of concern about how prepared they are to do so [14]. Our findings support the position that genetic specialists are currently best suited to assist patients with interpreting genetic test results. However, given the relatively modest differences in two of the three scenarios, providers may be able to accurately interpret genomic test results when specialists are unavailable, especially related to polygenic risk and pharmacogenomics. This may be because clinicians are trained to apply multifactorial clinical risk factors to patient decision making and use clinical information such as allergies and renal function to inform medication use. This is in contrast to the rare genetic disease scenario where non-genetic providers are less likely to have extensive experience. Based on the survey results, specialists are more confident and feel better prepared to perform genomic test interpretation in comparison to primary care providers, although the relatively small number of providers surveyed suggests that additional investigation is warranted.

Seeing age as a predictor in our regression analysis is not terribly surprising. It has been 15 years since the initial completion of the Human Genome project, and its impact has been immense. While medical education curricula are being adapted to include more genomic training, this has only started in earnest over the past ten years [36]. Physicians who completed their medical degree prior to 2007 may have had limited exposure to genetics education compared to those who graduated after 2007. Continuing education (CE) programs can help to increase knowledge in this topic. However, if genetics was excluded during specialization training, many providers may not prioritize taking CEs in genetics-based topics. According to adult learning theory, education is most effective when there is a recognized need to learn a subject and there is a desire to learn it [51, 52]. Therefore, those who lack exposure to genetics may have difficulty recognizing the benefits of taking genetic-based CEs, and thus will fail to seek them out. This is important given that the current study found previous experience discussing these kinds of reports was a factor in predicting higher interpretation scores. Those who had patients bring in reports tended to do better than those who have not. This supports the need for more exposure to genetics during medical school and residency programs.

Of particular note is the higher level of trust indicated by the providers group in comparison to the specialists, even though the specialists felt much more prepared to discuss results with patients. When evaluating the impact of the trustworthiness of DTC-GTs on the regression analysis, having a degree of skepticism may have helped. As one participant put it “whether I think the data is accurate, the answer is generally yes, but if you were asking do I think the descriptions and recommendations are comprehensive, the answer is no”. The provider group was generally more trusting of the tests, with less than 10% of the providers answering in the negative (untrustworthy or highly untrustworthy). This is in contrast to the 38.4% of specialist who had a negative level of trust. The specialists were also much less likely to indicate “highly trustworthy” (1.3%) than the providers who had selected that option (7.1%). Those who selected either “neutral” or “trustworthy” comprised the majority of both group (83.8% of the providers, 74.9% of the specialists, Fig. 3). In order to contextualize this properly, one has to consider the exponential pace at which genetic discoveries are occurring and thus are opening more frontiers to be explored than we have answers to. Consider that a recent study found that the average person has 54 genetic mutations that should be considered lethal, but don’t appear to harm their health. Under closer examination of this finding, it is hypothesized these mutations are errors in misclassification in the literature and/or in databases instead of genotyping error [53]. This discovery was uncovered via the Exome Aggregation Consortium (ExAC) database [54], which houses “60,706 unrelated individuals sequenced as part of various disease-specific and population genetic studies”. In 2016, Lek et al. also found 192 genetic variants that were thought to be pathogenic, which turned out to be relatively common. ExAC has provided much needed population level allele frequency data that has helped to reduce the bias related to affected family studies. This underscores how our comprehension of human genetics is still evolving, which should instill a sense of caution. As put by Dr. Isaac Kohane at Harvard Medical School “We really have a perfect storm of insufficient data and insufficient competence” in regards to physicians interpreting DTG-GT results [55]. Hence, when evaluating the level of trust that appears appropriate for DTC-GT, we can apply the Russian proverb popularized by Ronald Reagan, “trust, but verify”, an approach that parallels current FDA policies [56].

The high level of correct interpretation seen in the diabetes and drug risk is plausibly attributed to familiarity with these topics in these groups of providers and specialists. Scenario 2 provided a unique contrast, since COMPASS™is not a DTC-GT product. The HMNS7B scenario is typically encountered with less frequency as it represents a rare genetic disease and proved more challenging than the other scenarios. As a result, interpretation rates started to diverge between the two and we witnessed a drop in overall interpretation accuracy. We attribute part of these results as an artifact of the survey design. Our survey presented screenshots of reports, which are closer emulations of printed-paper reports than a more common web-based format. This provided participants with a limitation in the way they could interact and explore the data. Some specialists expressed frustration with this limitation in the free text feedback. A common refrain was the expressed desire for more information to assist in interpreting scenario 2, which was limited due to the static, non-interactive design of the scenarios. When evaluating the HMN7B report one participant expressed concern that it “never said how variant was classified with clarity”[sic]. The desire to delve deeper into the report and research the clinical significance of the variant in ClinVar [57] was expressed by several specialists. One participant commented that it was “alarming how much information the DTC reports give without giving any sort of context of the nuances that go into interpreting results”, several others also mirrored this concern about patient comprehension given the complexity and high reading level of some of the results. As designed, the COMPASS reports would be integrated into the electronic health record, with the ability to dynamically interact with the findings and provide links to help explain the decision process behind the reports. However, a key take away here is highlighting the nuance and challenge in presenting infrequent or rare genomic results, particularly when results are not straightforward. Specialists demonstrated heightened levels of scrutiny as demonstrated by their trustworthiness answers and feedback from scenario 2. The provider group appears to be more willing to accept genetic testing results at face value.

This creates a challenge for developing mechanisms to provide results back to clinicians in a meaningful fashion. Informatics tools for genetic consultations need to correctly highlight relevant information, preferably in an interactive format, and provide actionable options to its end users. However, not all genetic test results are easily interpreted, and determining the clinical relevancy of many genetic variants is an ongoing challenge as evidenced by the Clinical Genome Resource (ClinGen) project [58]. Heritability (the variance in phenotypic traits between individuals and a population) can have a large confounding impact on clinical decisions. In the case of well annotated variants associated with high probabilities of developing aggressive and often fatal cancers, such as *BRCA1* and *BRCA2* and hereditary breast and ovarian cancer, clinical decisions are fairly clear. However, in cases where the relevance is less clearly defined, there is a potential for greater uncertainty for interpretation. As mentioned earlier by a participant, there is a “nuance” to interpreting these tests. Understanding the methodologies and research cited to support the results presented appears to be desired by some specialists. This is information that may be overwhelming and less useful to patients who are not equipped to properly critique this data. Hence, it can be a precarious balancing act of distilling the information to the right level, which proves meaningful to both patient and providers.

Previous studies have examined the levels of DTC-GT customer comprehension of genetic results, and have found 72-79% were able to correctly interpret DTC-GTs (4-6) [4–6]. By sampling some of the same questions as described previously in the Ostergren study [6] (scenarios 1 & 3), we were able to compare our results with that study. Of this subset of scenarios, their study reported 81.8% of DTC customers surveyed were able to properly interpret their scenario questions. For the same four questions, 91.3% of the combined healthcare cohort (n=264) correctly interpreted them, which was a difference in proportion of 0.095, *p* <.001. One distinction to keep in mind is that customers of DTC-GT have been found to be more highly educated, and earn more than the median income in the US [4, 6]; therefore comprehension scores may dip when we move away from people who have both the disposable income and are curious enough to purchase DTC-GTs and we start to see a closer reflection of the public at large.

There are some limitations of this study, which warrant discussion. Response rate for the survey were low (9.1%), leading to a potential non-response bias. Two factors suspected to account for the low response rate include survey length (15 minutes to finish on average supported by the high abandonment rate) and lack of financial compensation for completions, which is a common practice to increase physician response rate on surveys. Balancing the number of questions to include in the survey with the length of the survey was a challenge but providing participants exposure to a variety of genomic results (disease risk, symptoms testing, and pharmacogenomics) was deemed important. Future work on this topic could be improved by increasing the sample size of primary care providers and clinical geneticists. The number of primary care providers sampled for this survey is small in comparison to the total number employed in the United States. In 2010, the were an estimated 209,000 primary care physicians, 56,000 nurse practitioners, and 30,000 physician assistants [40]. Additional work to improve the response and completion rates, while supporting diverse DTC-GT scenarios is warranted. When running the regression model to predict those who scored three or less on the interpretation task, it should be pointed out that very few participants selected the “highly untrustworthy” option (n=3), the vast majority who were wary of the tests elected to use “untrustworthy” (*n*=44). Having only three responses may not extrapolate the finding to the wider population of healthcare providers.

## Conclusions

The findings of this study indicate that specialists remain the best group to assist patients with DTC-GT, however, primary care providers may still provide accurate interpretation of test results when specialists are unavailable. The emerging challenge is how to tackle the infrequent or rare genomic results. Tasking specialists to address those results with non-specialist providers assisting with the more common cases is one solution. This is being actively explored by the Consent & Disclosure Recommendations (CADRe) working group of ClinGen [59]. The association of age as a predictor for correct interpretations suggests that younger providers are better prepared to take advantage of the new data being generated by precision medicine. There is an opportunity for developing training approaches to help close any gaps that appear due to training as genomic data begins to be integrated into EHRs. It is also encouraging that there was a positive association between prior experience consulting DTC-GT patients and interpretation scores. This suggests that increased exposure to these tests may lead providers to seek additional information to aid in interpretation and counseling further developing the skill to interpret them. This will help develop trust in these reports as an effective tool in patient consultations. Precision medicine is still in its infancy; however, our findings indicate that both providers and specialist are positioned to be able to take advantage of the promise of precision medicine as it matures and becomes more commonplace.

## Supplementary information

**Additional file 1** Survey instrument. Copy of the survey used in this study.

## Abbreviations

AANP: American association of nurse practitioners
AAPA: American academy of physician assistants
ACMG: American board of medical genetics and genomics
ACMGG: American college of medical genetics and genomics
AHRQ: Agency for healthcare research and quality
AMIA: American medical informatics association
CADRe: Consent and disclosure recommendations working group
CE: Continuing education
ClinGen: Clinical genome project
CSER WG: Clinical sequencing exploratory research consortium genetic counseling working group
CSER: Clinical sequencing exploratory research consortium
DTC-GT: Direct-to-Consumer genetic test
EHR: Electronic health record
eMERGE: electronic MEdical records and GEnomics network
ExAC: Exome aggregation consortium database
NSGC: National society of genetic counselors
PCP: Primary care provider
PMI: Precision medicine initiative
SGIM: Society of general internal medicine
