# Supplementary material for: Are providers prepared for genomic medicine: interpretation of Direct-to-Consumer genetic testing (DTC-GT) results and genetic self-efficacy by medical professionals
Source: BMC Health Serv Res. 2019 Nov 25;19:844. doi: 10.1186/s12913-019-4679-8 (PMC6876107; doi:10.1186/s12913-019-4679-8)
Supplement: Supplementary file 1 — Additional file 1 Survey instrument. Copy of the survey used in this study. [file 12913_2019_4679_MOESM1_ESM.pdf]

Benchmarking genetic testing interpretation and self efficacy of medical professionals

Informed Consent

This survey is best suited to be completed on a tablet or a computer.
In order to proceed with this survey, please read below:

Dear Sir or Madam,

Thank you for your interest in my study. I am a doctoral candidate at the University of Nebraska pursuing a Biomedical Informatics degree, and I am researching precision medicine. As part of my dissertation, I am looking to capture a snapshot of how prepared physicians and other health professionals are to address the genomic components of precision medicine. Precision medicine aims to add additional medical data like patient environmental exposure, lifestyle, and genetics to enhance overall care. This particular survey will be anonymous, and no identifying information will be collected (no names, IP addresses, or email). It will consist of **33 questions** (34 including the consent question) aiming to establish demographics (7 questions), genetic self-efficacy (6 questions), three patient genetic scenarios to interpret (11 questions total), and opinions on DTC genetic tests and Data sharing (5 questions). The genetic scenarios will present sample test results from a couple of direct-to-consumer genetic testing sites and a clinical genetic results navigation tool. You will have an opportunity to review excerpts from those reports and then answer a few questions about them. **It is expected to take around 15-20 minutes to complete the survey** If this is something you are interested in helping me with, please acknowledge your consent by clicking the "*Yes, I consent to participate in this study*". If for any reason, you decide to decline, you can click the "*No, I do not consent to participate*", and the survey will close out.

The IRB for this study (206-16-EX) was approved by the University of Nebraska Medical Center IRB. Pertinent questions or concerns about the research, research participants' rights, and/or research-related injuries to participants should be directed to the UNMC IRB irbora@unmc.edu, (402) 559-6463, unmc.edu/irb/.

Questions about this research should be addressed to Scott McGrath,(402) 577-0501, smcgrath@unomaha.edu or my dissertation committee chair, Dr. Kiran Bastola at dkbastola@unomaha.edu.

Thank you for your time,

Scott McGrath, MS
Biomedical Informatics Doctoral Candidate
University of Nebraska at Omaha
(402) 577-05071 - smcgrath@unomaha.edu

1. Study inclusion requirements are as follows:

- 19 years or older
- Hold a post-graduate or professional degree (i.e. MD, MS in genetic counseling, etc.)
- Consent to be included in this study

○ Yes, I consent to participate in this study

○ No, I do not consent to participate

Benchmarking genetic testing interpretation and self efficacy of medical professionals

Demographics

2. What is your age? (you can use the slider or enter it directly in the box to the right)

19 50 100

3. Gender

- Male
- Female
- I do not wish to self identify

4. Race

- **Hispanic or Latino**: persons of Mexican, Puerto Rican, Cuban, Central or South American, or other Spanish culture or origin, regardless of race
- **White** (Not Hispanic or Latino): persons having origins in the original people of Europe, North Africa or the Middle East
- **Black or African American** (Not Hispanic or Latino): persons having origins in the black racial groups of Africa
- **Native Hawaiian or other Pacific Islander** (Not Hispanic or Latino): persons having origins in the peoples of Hawaii, Guam, Samoa, or other Pacific Islands
- **Asian** (Not Hispanic or Latino): persons having origins in the original peoples of the Far East, SE Asia or the Indian Subcontinent, including, for example, Cambodia, China, India, Japan, Korea, Malaysia, Pakistan, Philippine Islands, Thailand, & Vietnam
- **American Indian or Alaskan Native** (Not Hispanic or Latino): persons having origins in the original peoples of North or South America, and who maintain cultural identification through tribal affiliation or community attachment
- **Two or More Races** (Not Hispanic or Latino): persons who identify with more than one of the above races
- **I do not wish to self-identify**

5. Medical Specialty

- Medical/Clinical Geneticist
- Internist
- Genetic Counselor
- Family Medicine
- Primary Care Physician
- Other (please specify)

6. How many years have you been practicing medicine?

1 25 50

7. What zip code do you primarily work in?

8. Which best describes your workplace?

- Solo practice
- Clinic
- Group practice
- Integrated Healthcare Delivery System
- Employed Physician practice
- Locum Tenens
- Hospital
- Other (please specify)

Genetic self-efficacy

Self-efficacy is the belief in one's ability to accomplish a task. The following questions aim to establish self-efficacy on tasks involving genetics.

9. I am able to understand information about how genes can affect my patients' health.

- ○ Strongly disagree
- ○ Disagree
- ○ Somewhat disagree
- ○ Somewhat agree
- ○ Agree
- ○ Strongly agree

10. I am confident in my ability to understand information about genetics.

- ○ Strongly disagree
- ○ Disagree
- ○ Somewhat disagree
- ○ Somewhat agree
- ○ Agree
- ○ Strongly agree

11. I have a good idea about how genetics may influence risk for disease generally.

- ○ Strongly disagree
- ○ Disagree
- ○ Somewhat disagree
- ○ Somewhat agree
- ○ Agree
- ○ Strongly agree

12. I have a good idea about how genetic make-up might affect my patients' risk for disease.

- ○ Strongly disagree
- ○ Disagree
- ○ Somewhat disagree
- ○ Somewhat agree
- ○ Agree
- ○ Strongly agree

13. I am able to explain to others how genes affect one’s health.

- Strongly disagree
- Disagree
- Somewhat disagree
- Somewhat agree
- Agree
- Strongly agree

UNIVERSITY OF Nebraska Omaha

Benchmarking genetic testing interpretation and self efficacy of medical professionals

Genetic interpretation scenario 1

Please note these are representative results, current results may differ from what is depicted here.

The following is an example report from ***23andMe****. DTC genetic test companies offer results directly to consumers without physician involvement. The consumer may potentially take these results to their own personal physician for guidance.*

Please read each scenario, review the reports and answer the accompanying questions.

Scenario 1) Imagine your patient Dan received the following information about his risk for type 2 diabetes and has brought it in for you to review. Dan is a 35-year-old man of European ancestry who is 5 feet 8 inches tall and weighs 210 lbs. According to his Body Mass Index, he is considered to be obese. Dan does not currently have type 2 diabetes.

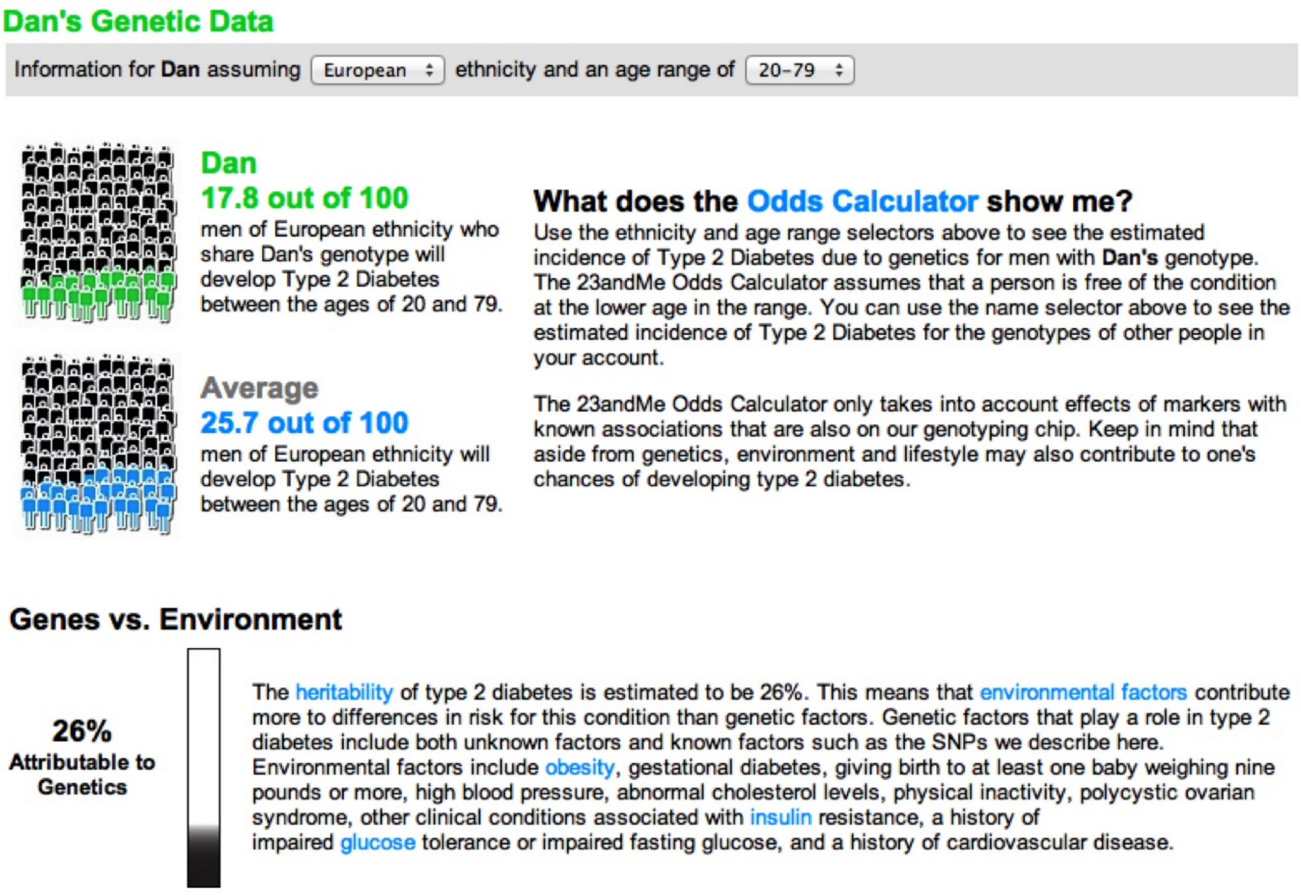

Dan's Genetic Data

Information for **Dan** assuming European ethnicity and an age range of 20–79

Dan

17.8 out of 100

men of European ethnicity who share Dan's genotype will develop Type 2 Diabetes between the ages of 20 and 79.

Average

25.7 out of 100

men of European ethnicity will develop Type 2 Diabetes between the ages of 20 and 79.

What does the Odds Calculator show me?

Use the ethnicity and age range selectors above to see the estimated incidence of Type 2 Diabetes due to genetics for men with **Dan's** genotype. The 23andMe Odds Calculator assumes that a person is free of the condition at the lower age in the range. You can use the name selector above to see the estimated incidence of Type 2 Diabetes for the genotypes of other people in your account.

The 23andMe Odds Calculator only takes into account effects of markers with known associations that are also on our genotyping chip. Keep in mind that aside from genetics, environment and lifestyle may also contribute to one's chances of developing type 2 diabetes.

Genes vs. Environment

26%
Attributable to Genetics

The heritability of type 2 diabetes is estimated to be 26%. This means that environmental factors contribute more to differences in risk for this condition than genetic factors. Genetic factors that play a role in type 2 diabetes include both unknown factors and known factors such as the SNPs we describe here. Environmental factors include obesity, gestational diabetes, giving birth to at least one baby weighing nine pounds or more, high blood pressure, abnormal cholesterol levels, physical inactivity, polycystic ovarian syndrome, other clinical conditions associated with insulin resistance, a history of impaired glucose tolerance or impaired fasting glucose, and a history of cardiovascular disease.

14. Based on these GENETIC results, what are Dan’s chances of developing diabetes compared to the average man of his age and ethnicity?

- ◯ Much higher
- ◯ Somewhat higher
- ◯ About the same
- ◯ Somewhat lower
- ◯ Much lower

15. Based on these GENETIC results, do you think that Dan:

- ◯ Definitely will develop diabetes
- ◯ Probably will develop diabetes
- ◯ Probably will NOT develop diabetes
- ◯ Definitely will NOT develop diabetes

16. Which of the following is a true statement about Dan’s risk of diabetes?

- ◯ Dan’s obesity is an IMPORTANT risk factor for diabetes regardless of his genetic results.
- ◯ Dan’s obesity is LESS of a risk factor for diabetes because of his genetic results
- ◯ Dan’s obesity is NOT a risk factor for diabetes

17. How helpful was the material provided in determining Dan’s disease risk?

	Very Unhelpful	Unhelpful	Neutral	Helpful	Very Helpful
Visualizations (shaded group, filled bar graph)	◯	◯	◯	◯	◯
"Odds calculator" text	◯	◯	◯	◯	◯
"Genes vs. Environment" text	◯	◯	◯	◯	◯

18. Please rank the elements from this scenario that you feel would be most valuable to include in patient records (for example, in their EHR), if this type of testing became more widespread?

You can drag the items into order or select the items from the drop-down box (1 = most valuable - 3 = least valuable)

- Visualizations (shaded group, filled bar graph)
- "Odds calculator" text
- "Genes vs. Environment" text

19. In your opinion, how concerned should Dan be about his results?

- Very concerned
- Somewhat concerned
- Feelings unchanged
- Somewhat less concerned
- A lot less concerned

Genetic interpretation scenario 2

Please note these are representative results, actual results may differ.

*The following is an excerpt from **Geisinger's Compass Genome Report**. Please read the scenario, review the reports and answer the accompanying questions.*

Scenario 2) Imagine your patient Erin received the following whole genome sequencing test results highlighting DCTN1 as a possible cause for her symptoms. She has brought in the report for you to review. Erin is a 48-year-old woman,with three children.

Report Overview

It is estimated that the human body contains 20,000 genes. We currently understand the function of only about 3,000 genes and how certain types of variants affect this function. Some genetic variants can have no effect at all, some may even be helpful, and others may be harmful. We suggest that you stay in contact with your healthcare provider and/or genetics professional at least once a year to learn if there is any new information related to your Whole Genome Sequencing test results.

Reason for Testing

Whole genome sequencing testing was ordered to identify a possible genetic cause for your symptoms. Your symptoms were reported to include: **Dysphagia or feeding difficulty, Muscular atrophy or hypoplasia, Recurrent exacerbations**

What is included in this report?

Primary Findings **1**

Was at least one relevant genetic variant found? **Yes**

DCTN1 ☐

Summary of Results

A possible genetic cause for symptoms was found with a potential diagnosis of HMN7B: distal hereditary motor neuronopathy VIIB. It is important to talk with your doctor about the meaning of these results.

Information about the DCTN1 Gene

What does this gene do?

The DCTN1 gene provides instructions for making a protein called dynactin-1. At least two different versions of this protein are produced in cells. Both versions of the dynactin-1 protein interact with several other proteins to form a group (a complex) of proteins called dynactin. This complex plays a critical role in cell division and the transport of materials within cells. To carry out these roles, the complex attaches (binds) to a protein called dynein, which acts as a motor, and also binds to a track-like system of small tubes called microtubules. The dynactin complex, dynein, and microtubules work together like a conveyer belt to move materials within cells. Researchers believe that the dynactin complex is particularly important for the proper function of axons, which are specialized extensions of nerve cells (neurons). Axons transmit impulses from nerve to nerve and from nerves to muscles. Axons can be quite long; some are more than 3 feet in length. The dynactin complex is a critical part of a rapid transport system that supplies axons with materials to keep them healthy and functioning efficiently. (Taken from Genetics Home Reference)

What variant(s) were found?

- ENST00000361874:c.2432C>G:p.Pro811ArgG>C;

How do variants in this gene cause health problems?

Pathogenic variants in the DCTN1 gene disrupt the function of the dynactin complex leading to a number of different conditions that affect the brain, nerves and muscles (neuromuscular system). Depending on the type of variant, certain features may be more prominent than others.

Distal hereditary motor neuronopathy type VIIB: At least one DCTN1 gene variant appears to cause a nervous system disorder called distal hereditary motor neuronopathy type VIIB. Signs and symptoms of this disorder first appear in early adulthood and include breathing difficulties and progressive weakness of muscles in the face and hands. Muscle weakness in the feet and legs develops later.

Perry syndrome: Some variants in DCTN1 cause this condition. Signs and symptoms can include a pattern of movement abnormalities known as parkinsonism, behavior changes, weight loss, and abnormally slow breathing (hypoventilation).

Amyotroic Lateral Sclerosis: Several variants in the DCTN1 gene may increase the risk of developing amyotrophic lateral sclerosis (ALS), a condition characterized by progressive movement problems and muscle wasting. As opposed to the most severe form of ALS (also known as Lou Gehrig's Disease), this form seems to be milder and more slowly progressive. Atrophy (wasting) of the muscles of the hand may be the first sign of this condition.

Are the variants the cause of the symptoms?

This variant is possibly the cause of your symptoms because: Pathogenic variants in the DCTN1 gene can cause several different conditions that have relevance to your presentation as noted in the indication for testing and summary of findings.

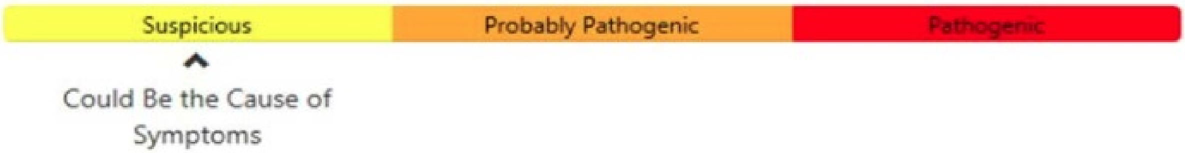

Understanding the Diagnosis

This table summarizes the most common and medically important symptoms associated with HMN7B: distal hereditary motor neuropathy VIIB. There are a range of symptoms associated with this condition. Each person with HMN7B: distal hereditary motor neuropathy VIIB will have his or her own unique combination of the symptoms listed. Some symptoms are more common than others and are indicated this way:

How many people with HMN7B: distal hereditary motor neuropathy VIIB are expected to have this symptom?

Also, keep in mind that our understanding and management of HMN7B: distal hereditary motor neuropathy VIIB will change as researchers continue to study this condition. It is not possible to predict what new types of treatments and interventions will be available in the future through advances in medical science.

Table Key	
None	None or NA
Few	less than or equal to 30%
Some	more than 30%
Most	more than 85%

By what age do most people with HMN7B: distal herediarty motor neuropathy VIIB have this symptom?

Symptom	Birth	1 month	3 months	6 months	1 year	3 years	6 years	10 years	15 years	25 years	40 years	60 years	80 years
Emergence over years	NA	NA	NA	NA	NA	NA	NA	NA	Few	Some	Most	Most	Most
Hypotonia	NA	NA	NA	NA	NA	NA	NA	NA	Few	Some	Some	Some	Some
Weakness, significant	NA	NA	NA	NA	NA	NA	NA	NA	Few	Some	Some	Some	Some
Muscular atrophy or hypoplasia	NA	NA	NA	NA	NA	NA	NA	NA	Few	Some	Some	Some	Some
Facial weakness	NA	NA	NA	NA	NA	NA	NA	NA	Few	Some	Some	Some	Some
Respiratory difficulty	NA	NA	NA	NA	NA	NA	NA	NA	Few	Few	Some	Some	Some
Dysarthria or abnormal sound character	NA	NA	NA	NA	NA	NA	NA	NA	Few	Few	Some	Some	Some
Upper body predominance to findings	NA	NA	NA	NA	NA	NA	NA	NA	Few	Few	Few	NA	NA
**Symptoms that can be detected only by a special test**													
DCTN1 gene mutation (monoallelic)	Most	Most	Most	Most	Most	Most	Most	Most	Most	Most	Most	Most	Most
Laryngoscopy: vocal cord paresis	NA	NA	NA	NA	NA	NA	NA	NA	Few	Some	Some	Some	Some

20. Based on these GENETIC results, do you think that Erin:

- ◯ Definitely has HMN7B: distal hereditary motor neuropathy VIIB
- ◯ Probably has HMN7B: distal hereditary motor neuropathy VIIB
- ◯ Probably does NOT have HMN7B: distal hereditary motor neuropathy VIIB
- ◯ Definitely does NOT have HMN7B: distal hereditary motor neuropathy VIIB

21. Which is a true statement for Erin's risk of developing symptoms related to HMN7B

- ◯ Erin is not at risk for developing symptoms associated with HMN7B
- ◯ Most of the symptoms related to HMN7B would have been present for the past 20 years or so
- ◯ Most of the symptoms related to HMN7B would have been present for the past 10 years or so
- ◯ Most of the symptoms related to HMN7B will emerge in the future

22. How helpful was the material provided in determining if Erin's symptoms are associated with the genetic test result?

	Very Unhelpful	Unhelpful	Neutral	Helpful	Very Helpful
"Report Overview" text block	◯	◯	◯	◯	◯
"Reason for testing" text block	◯	◯	◯	◯	◯
"What does this gene do?" text block	◯	◯	◯	◯	◯
"What variant(s) were found?" text	◯	◯	◯	◯	◯
"How do variants in this gene cause health problems?" text block	◯	◯	◯	◯	◯
"Are the variants the cause of the symptoms? " text and graphic	◯	◯	◯	◯	◯
"Understanding the Diagnosis" text block	◯	◯	◯	◯	◯
"Understanding the Diagnosis " table	◯	◯	◯	◯	◯

23. Please rank the elements from this scenario that you feel would be most valuable to include in patient records (for example, in their EHR), if this type of testing became more widespread?

You can drag the items into order or select the items from the drop-down box (1 = most valuable - 8 = least valuable)

- "Report Overview" text block
- "Reason for testing" text block
- "What does this gene do?" text block
- "What variant(s) were found?" text
- "How do variants in this gene cause health problems?" text block
- "Are the variants the cause of the symptoms?" text & graphic
- "Understanding the Diagnosis" text
- "Understanding the Diagnosis" table

24. In your opinion, how concerned should Erin be about her results?

- ◯ Very concerned
- ◯ Somewhat concerned
- ◯ Feelings unchanged
- ◯ Somewhat less concerned
- ◯ A lot less concerned

Genetic interpretation scenario 3

Please note these are representative results, actual results may differ.

*The following are example reports from **Pathway Genomics**. DTC genetic test companies offer results directly to consumers without physician involvement. The consumer may potentially take these results to their own personal physician for guidance. Please read each scenario, review the reports and answer the accompanying questions.*

Imagine your patient Frank takes a statin drug called simvastatin to reduce his cholesterol level. Frank receives the following drug response results:

DRUG RESPONSE

PATHWAY GENOMICS®

Statins : Myopathy

Results

Patient has a genetic marker that significantly increases the risk of statin-induced myopathy (muscular pain and damage). About 5-10% of patients taking statins experience myopathy. The potential risk of myopathy should be weighed against the benefits of statins. If the patient is being treated with a statin, muscle symptoms should be carefully monitored. A reduced statin dose or non-statin therapeutic could be considered.

About this medication

Statins (atorvastatin, fluvastatin, lovastatin, pitavastatin, pravastatin, rosuvastatin, simvastatin) are a type of widely prescribed cholesterol-lowering medicines. They block the production of cholesterol in cells by inhibiting a certain enzyme that is critical in the synthesis of cholesterol.

We evaluated the following markers

Gene	Marker	**Your Genotype**
SLCO1B1	rs4149056	T/C

Genetics of this response

The major adverse effect of statins is pain and damage in the skeletal muscles (myopathy). About 5-10% of patients taking statins experience muscle pain (myalgia). A small portion of patients may develop more severe symptoms including muscle weakness, muscle cramps, myositis (inflammation of muscles, may be accompanied by increased creatine kinase levels in the blood), and the rare but potentially lethal rhabdomyolysis. In rare cases, myalgia and creatine kinase elevations persist after statin withdrawal. When rhabdomyolysis occurs, skeletal muscles rapidly break down, releasing large quantities of muscle cell contents into the blood. Some of those contents, such as myoglobin, cannot be properly processed by the kidneys and may lead to acute renal failure and death. In randomized, controlled trials, reported incidence of statin-induced myopathy ranges from 1.5% to 5.0%. The rate of statin-induced rhabdomyolysis is approximately 0.1 to 0.2 cases per 1000 person-years. The risk of myopathy varies with the type of statin and is dose-related. The incidence of myopathy while taking 80 mg simvastatin daily is more than 25 times the incidence of a daily dose of 20 mg.

25. Based on his statins drug response results, what are Frank's chances of myopathy while taking statin therapy?

- ◯ Higher than average
- ◯ Lower than average
- ◯ About average

26. How helpful was the material provided in determining Frank's disease risk?

	Very Unhelpful	Unhelpful	Neutral	Helpful	Very Helpful
Initial block of information: "Results"	◯	◯	◯	◯	◯
"About this medication" text	◯	◯	◯	◯	◯
"We evaluated the following" table	◯	◯	◯	◯	◯
"Genetics of this response" text	◯	◯	◯	◯	◯

27. Please rank the elements from this scenario that you feel would be most valuable to include in patient records (for example, in their EHR), if this type of testing became more widespread?

You can drag the items into order or select the items from the drop-down box (1 = most valuable - 4 = least valuable)

- Initial block of information: "Results"
- "About this medication" text
- "We evaluated the following" table
- "Genetics of this response" text

28. If you had been presented results from a patient as depicted in the scenarios, overall, how trustworthy would you rate the information?

- Highly trustworthy
- Trustworthy
- Neutral
- Untrustworthy
- Highly untrustworthy

29. How satisfied were you with the reports from the various scenarios in helping you understand the case presented?

	Very Dissatisfied	Dissatisfied	Unsure	Satisfied	Very Satisfied
23andMe (Scenario #1)					
COMPASS (Scenario #2)					
Pathway Genomics (Scenario #3)					

30. Have you had any patients bring in DTC genetic test results to share with you (like those depicted in the scenarios)? *If yes, please enter an approximate number of how many patients in the box.*

- No
- Yes (please enter an approximate number in the box below)

31. How prepared do you think you are for discussing genetic testing results with your patients?

- ◯ Very Unprepared
- ◯ Somewhat Unprepared
- ◯ Nuetral
- ◯ Somewhat Prepared
- ◯ Well Prepared

In order optimize the benefits of genetic testing for clinical practice, data sharing will be crucial. This final section will present questions about sharing of health information for research.

32. *For this question, please insert each answer listed below into the blank of the bolded question and supply your answer.* In thinking about a large study using information from **your** electronic health record (*not patient data*) that is sent electronically to health researchers, How much do you agree with each of the following statements, "**I would be comfortable if ____________________ had electronic access to my anonymous health information for research**."

	Strongly agree	Agree	Neutral	Disagree	Strongly disagree
a doctor's office	◯	◯	◯	◯	◯
a hospital	◯	◯	◯	◯	◯
an insurance company	◯	◯	◯	◯	◯
a state or local health agency	◯	◯	◯	◯	◯
a national government health agency	◯	◯	◯	◯	◯
a pharmaceutical company	◯	◯	◯	◯	◯
a biotechnology company	◯	◯	◯	◯	◯
a college or university	◯	◯	◯	◯	◯

33. How much do you agree or disagree with these statements:

	Strongly Agree	Agree	Neutral	Disagree	Strongly disagree
Research that could be beneficial to people's health is more important than protecting people's privacy.	○	○	○	○	○
The risk of possible loss of confidentiality of health information is greater than the benefit of health research.	○	○	○	○	○
An Individual's right to control use of their health information is more important than the possible benefits of health research.	○	○	○	○	○

Benchmarking genetic testing interpretation and self efficacy of medical professionals

Survey Exit

I know your time is valuable, so thank you for your help.

If you have any questions or concerns, please reach me at (402) 577-0501 or smcgrath@unomaha.edu.

34. (Optional) If you have any thoughts or insights you would like to share, please feel free to provide them here.
